# New Alphacoronavirus in *Mystacina tuberculata* Bats, New Zealand

**DOI:** 10.3201/eid2004.131441

**Published:** 2014-04

**Authors:** Richard J. Hall, Jing Wang, Matthew Peacey, Nicole E. Moore, Kate McInnes, Daniel M. Tompkins

**Affiliations:** Institute of Environmental Science and Research, Upper Hutt, New Zealand (R.J. Hall, J. Wang, M. Peacey, N.E. Moore);; Department of Conservation, Wellington, New Zealand. (K. McInnes); and Landcare Research, Dunedin, New Zealand. (D.M. Tompkins)

**Keywords:** alphacoronavirus, coronavirus, viruses, guano, metagenomics, bats, Mystacina tuberculata, New Zealand

## Abstract

Because of recent interest in bats as reservoirs of emerging diseases, we investigated the presence of viruses in *Mystacina tuberculata* bats in New Zealand. A novel alphacoronavirus sequence was detected in guano from roosts of *M. tuberculata* bats in pristine indigenous forest on a remote offshore island (Codfish Island).

Human settlement in New Zealand is relatively recent compared with that of many countries and extends back ≈800 years to when early Polynesian explorers first arrived (*1*). A wide variety of exotic flora and fauna have since been introduced, and major changes to the landscape and ecology have occurred, particularly once European settlers arrived 150 years ago. However, large areas of New Zealand that are representative of a prehuman state still remain, in particular offshore, islands such as Whenua hou (Codfish Island), which is situated off the southern coast of New Zealand. Before human settlement, only 3 species of terrestrial mammals were present, all of which were bats. Why nonvolant mammals have been absent is unknown, but this absence was probably caused by major extinction events and subsequent geographic isolation that prevented recolonization (*2**,**3*).

Only 2 bat species remain in New Zealand and both are considered vulnerable (www.iucnredlist.org/). The long-tailed bat (*Chalinolobus tuberculatus*) belongs to the family Vespertilionidae and is believed to have arrived from Australia ≈1 million years ago (*4*). The lesser short-tailed bat (*Mystacina tuberculata*) is the sole surviving member of the family Mystacinidae in New Zealand (*5*). *M. tuberculata* bats are believed to have diverged from other bat species ≈20 million years ago (*6*) and have lived in geographic isolation from other bat species until the arrival of *C. tuberculatus* bats (*3*). *M. tuberculata* bats also exhibit one of the widest feeding ranges of any bat species and occupy a niche similar to rats and mice because these bats can walk on the ground by using their wings (*7*).

Little is known about the microorganisms present in these bats. One study found no evidence of pathogenic bacteria or lyssaviruses in them, but reported a *Sarcocystis* sp. (*8*). Given the recent intense interest in bats as a reservoir of emerging diseases and the advent of high-throughput sequencing as a virus discovery tool, we investigated the presence of viruses in *M. tuberculata* bats.

## The Study

Four bat guano samples were collected from known roost sites on the remote offshore island of Whenua hou (Codfish Island) (46°47′S, 167°38′E), which is situated at the southern coast of New Zealand. This small island is heavily forested and largely unmodified by humans. It has special conservation status for the protection of endangered species, and public access is not permitted (*9*).

Bat guano was held at 4°C during transport (<48 h), resuspended in 2 mL phosphate-buffered saline, and subjected to centrifugation at 6,000 × *g* for 5 min. RNA was extracted from 400 µL of supernatant by using the iPrep PureLink Virus Kit (Life Technologies, Carlsbad, CA, USA) and eluted into 50 µL reverse transcription PCR molecular-grade water (Ambion, Austin, TX, USA). Metagenomic sequencing was then conducted for 1 of the samples by using an Illumina MiSeq Instrument (New Zealand Genomics Ltd., Massey Genome Service, Massey University, Palmerston North, New Zealand) after a series of steps involving DNase I treatment, reverse transcription, multiple displacement amplification (QIAGEN, Valencia, CA, USA), and Illumina TruSeq library preparation (New Zealand Genomics, Ltd.).

A total of 10,749,878 paired-end sequence reads of 250 bp were generated and assembled into contigs by using Velvet 1.2.07 (*10*). Assembled contigs were searched for viral sequence by comparison to the nonredundant nucleotide database in Genbank (downloaded March 2013) by using the nucleotide basic local alignment search tool (National Center for Biotechnology Information, Bethesda, MD, USA). Forty-six contigs showed similarity to known genus *Alphacoronavirus* sequences (Table). (Raw sequence data are available on request from the authors.)

**Table:** Summary of BLASTn output for contigs from bat guano that show identity to alphacoronaviruses, New Zealand*

Alphacoronavirus gene	No. contigs matching alphacoronaviruses	Range of contig lengths, bp	Highest scoring BLASTn hit recorded against alphacoronaviruses	
e-value	Nucleotide identity (%)†
Open reading frame 1ab	33	182–1054	8 × 10^−93^	581/828 (77)
Spike protein	5	580–1629	1 × 10^−19^	362/551 (66)
Matrix	4	251–840	2 × 10^−99^	532/746 (71)
Nucleocapsid	4	536–890	6 × 10^−6^	79/109 (72)

*BLASTn, nucleotide basic local alignment search tool (http://blast.ncbi.nlm.nih.gov/Blast.cgi?PROGRAM=blastn&PAGE_TYPE=BlastSearch&LINK_LOC=blasthome). †No. nucleotides identical between query and database sequence.

**Figure 1:**
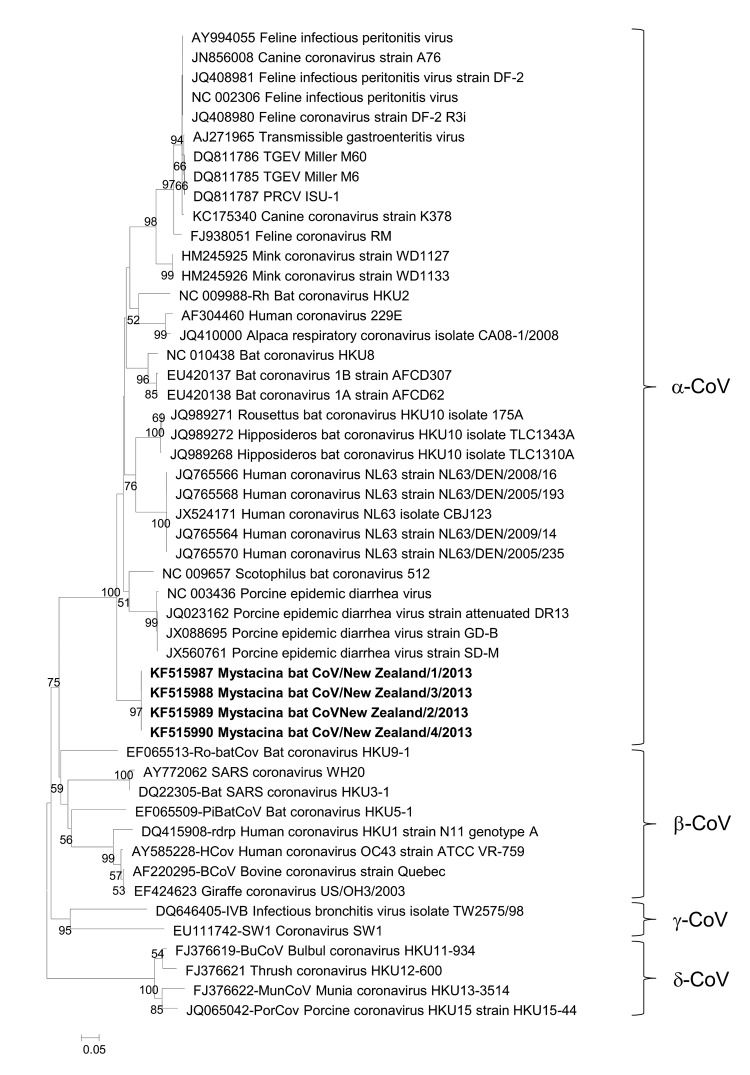
Phylogenetic tree showing genetic relatedness of RNA-dependent RNA polymerase amino acid sequences for *Mystacina* sp. bat coronavirus (CoV)/New Zealand/2013 (shown in **boldface**) with those of known coronaviruses. Evolutionary history was inferred for 183 informative amino acid sites by using the maximum-likelihood method based on the Whilan and Goldman model with gamma distribution in MEGA 5.05 software (www.megasoftware.net). Bootstrap values are calculated from 1,000 trees (only bootstrap values >50% are shown). Scale bar indicates nucleotide substitutions per site. TGEV, transmissible gastroenteritis CoV; PRCV, porcine respiratory CoV; SARS, severe acute respiratory syndrome.

**Figure 2:**
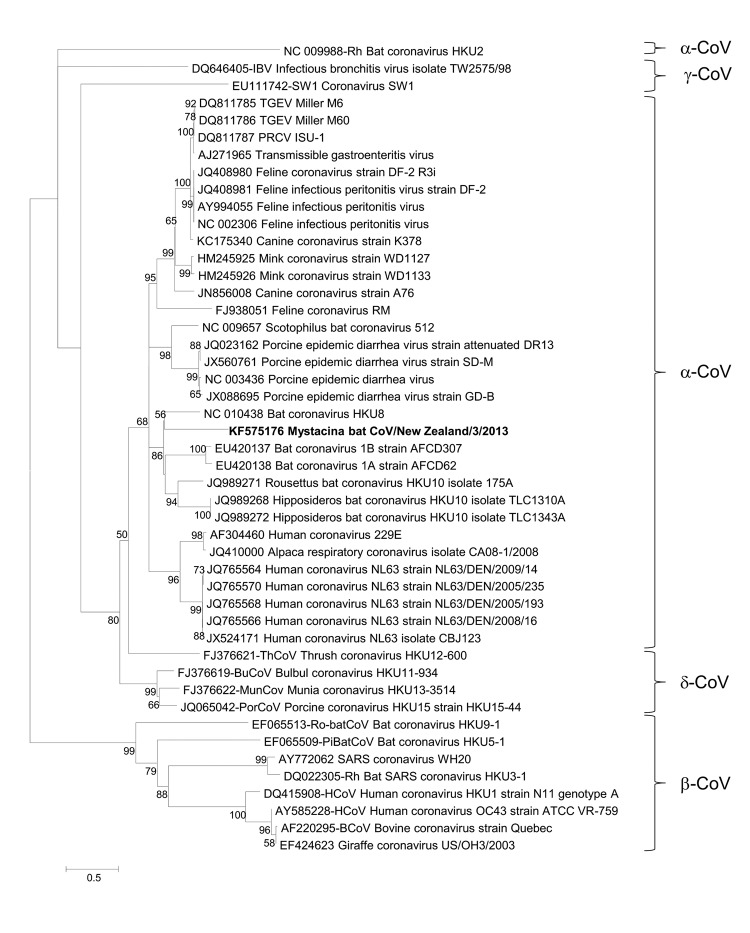
Phylogenetic tree showing genetic relatedness of spike protein amino acid sequence for *Mystacina* sp. bat coronavirus (CoV)/New Zealand/2013 (shown in **boldface**) with those of known coronaviruses. Evolutionary history was inferred for 492 informative amino acid sites by using the maximum-likelihood method based on the Whilan and Goldman + F model with gamma distribution and invariant sites in MEGA 5.05 software (www.megasoftware.net). Bootstrap values are calculated from 1,000 trees (only bootstrap values >50% are shown). Scale bar indicates nucleotide substitutions per site. TGEV, transmissible gastroenteritis CoV; PRCV, porcine respiratory CoV; SARS, severe acute respiratory syndrome.

Virus sequence from the genus *Alphacoronavirus* was confirmed in all 4 original guano samples by using a specific reverse transcription PCR based on the metagenomic data specific for 582 bp of the RNA-dependent RNA polymerase (*RdRp*) gene (GenBank accession nos. KF515987–KF515990). The *Rdrp* sequence was identical in all 4 guano samples, and the closest relative was bat coronavirus HKU8 (GenBank accession no. DQ249228; 79% nt identity, 426/542). Phylogenetic analysis was performed for *Rdrp* (Figure 1) and for the spike protein, as derived from metagenomic data (GenBank accession no. KF575176) (Figure 2). We propose that these data support identification of a new alphacoronavirus, which has been designated as *Mystacina* bat CoV/New Zealand/2013.

## Conclusions

The discovery of unknown coronaviruses provides information for a model of coronavirus evolution (*11*) and contributes to understanding the process of disease emergence, as in detection of Middle East respiratory syndrome coronavirus (*12*). The alphacoronavirus identified in this study from *M. tuberculata* bat guano is unique in respect to the extreme geographic and evolutionary isolation of the host bat species, which along with *C. tuberculatus* bats, has been separated from all other mammalian species for ≈1 million years.

The current estimate for a common ancestor for all coronaviruses is 8,100 bce (*13*). To be consistent with this estimate, *Mystacina* bat coronavirus would need to have been introduced to bats on Whenua hou within the past 800 years since humans first arrived on this island (given that the island had no other mammals before this time) (*11*) or is extant to modern alphacoronavirus phylogenetic radiation (genesis and expansion). Apart from humans, only 2 other terrestrial mammals have ever inhabited Whenua hou: the brushtail possum (*Trichosurus vulpecula*) and the Polynesian rat (*Rattus exulans*), both of which were eliminated from the island in the late 1980s (*11*); neither mammal has been reported as a host of alphacoronaviruses. Members of the genus *Alphacoronavirus* infect only mammals. Thus, an avian origin for this virus is unlikely.

An alternative theory of an ancient origin for all coronaviruses has recently been proposed that involves an alternative evolutionary molecular clock analysis, which places the most recent common ancestor many millions of years ago (*14*). The discovery of *Mystacina* bat CoV/New Zealand/2013 virus could lend support to such a theory; despite potentially millions of years of isolation, it has diverged relatively little from other extant alphacoronaviruses, as shown by the close relationship of the *Rdrp* and spike protein genes to those of other extant alphacoronaviruses (Figures 1, 2). An expanded survey for *Mystacina* bat coronavirus in mammals in New Zealand and subsequent characterization of viral genomes would provide further insights into the origin of coronaviruses.

No instances of human zoonotic disease from New Zealand bat species have been reported. Only a small number of conservation staff handle these bats, these staff use standard personal protective equipment and work practice. Staff are also offered prophylactic rabies vaccination as a precautionary measure, even though New Zealand is free from rabies. The genus *Alphacoronavirus* includes several human and animal pathogens, but on the basis of phylogenetic data in this study, it is not possible to estimate the risk posed by *Mystacina* bat coronavirus to human or animal health.

New Zealand is not considered a hot spot for emerging infectious diseases. This country is free from many human and animal diseases, such as rabies and foot-and-mouth disease, and infections with human arboviruses because of recent colonization by humans and strict biosecurity border controls (*15*). Thus, indigenous wildlife in New Zealand has generally been viewed as an almost sterile and unique biosphere. Given detection of this coronavirus, more thorough characterization of the ecology of viruses and other microorganisms in native wildlife should be considered to fulfill conservation needs and further safeguard human and domestic animal health against cross-species transmission.
